# Broadband scattering properties of articular cartilage zones and their relationship with the heterogenous structure of articular cartilage extracellular matrix

**DOI:** 10.1117/1.JBO.28.12.125003

**Published:** 2023-12-13

**Authors:** Iman Kafian-Attari, Ervin Nippolainen, Florian Bergmann, Akuroma George, Petri Paakkari, Arash Mirhashemi, Florian Foschum, Alwin Kienle, Juha Töyräs, Isaac O. Afara

**Affiliations:** a University of Eastern Finland, Department of Technical Physics, Kuopio, Finland; b Kuopio University Hospital, Diagnostic Imaging Center, Kuopio, Finland; c University of Ulm, Institute for Laser Technologies in Medicine and Meteorology, Ulm, Germany; d Kuopio University Hospital, Science Service Center, Kuopio, Finland; e University of Queensland, School of Information Technology, and Electrical Engineering, Brisbane, Queensland, Australia

**Keywords:** articular cartilage, tissue optical properties, scattering anisotropy factor, diffuse optical spectroscopy, polarized light microscopy

## Abstract

**Significance:**

Articular cartilage exhibits a zonal architecture, comprising three distinct zones: superficial, middle, and deep. Collagen fibers, being the main solid constituent of articular cartilage, exhibit unique angular and size distribution in articular cartilage zones. There is a gap in knowledge on how the unique properties of collagen fibers across articular cartilage zones affect the scattering properties of the tissue.

**Aim:**

This study hypothesizes that the structural properties of articular cartilage zones affect its scattering parameters. We provide scattering coefficient and scattering anisotropy factor of articular cartilage zones in the spectral band of 400 to 1400 nm. We enumerate the differences and similarities of the scattering properties of articular cartilage zones and provide reasoning for these observations.

**Approach:**

We utilized collimated transmittance and integrating sphere measurements to estimate the scattering coefficients of bovine articular cartilage zones and bulk tissue. We used the relationship between the scattering coefficients to estimate the scattering anisotropy factor. Polarized light microscopy was applied to estimate the depth-wise angular distribution of collagen fibers in bovine articular cartilage.

**Results:**

We report that the Rayleigh scatterers contribution to the scattering coefficients, the intensity of the light scattered by the Rayleigh and Mie scatterers, and the angular distribution of collagen fibers across tissue depth are the key parameters that affect the scattering properties of articular cartilage zones and bulk tissue. Our results indicate that in the short visible region, the superficial and middle zones of articular cartilage affect the scattering properties of the tissue, whereas in the far visible and near-infrared regions, the articular cartilage deep zone determines articular cartilage scattering properties.

**Conclusion:**

This study provides scattering properties of articular cartilage zones. Such findings support future research to utilize optical simulation to estimate the penetration depth, depth-origin, and pathlength of light in articular cartilage for optical diagnosis of the tissue.

## 1 Introduction

The behavior of light in biological tissues is governed by intrinsic fundamental and wavelength-dependent properties (optical properties), including absorption coefficient ($\mu_{a}$), scattering coefficient ($\mu_{s}$), extinction coefficient ($\mu_{t}=\mu_{s}+\mu_{a}$), scattering anisotropy factor (g), reduced scattering coefficient ($\mu_{s}^{\prime }$), and refractive index (n).^1^^–^^3^ The key factors determining the optical properties of biological tissues are chemical composition, concentration, size, distribution, and alignment of the tissue matrix’s biomolecules.^3^ Since these vary between biological tissues and within the same tissue due to biological or pathological processes, this variation often leads to differences in optical properties between healthy and pathological tissues. Thus, the optical properties of biological tissues have been adopted as biomarkers for the non-invasive screening of tissue pathologies in multiple organs, including muscles, brain, skin, heart, and breast.^1^

In musculoskeletal research, particularly relating to osteoarthritis, there is growing research interest in applying diffuse optical technologies for the diagnostic assessment of musculoskeletal tissues, particularly with a focus on early detection and characterization of degenerative diseases, such as osteoarthritis.^4^^–^^9^ One of the primary tissues of focus in musculoskeletal research is articular cartilage, the highly specialized connective tissue covering the ends of bones in articulating joints. Articular cartilage enables joint lubrication and transmission of load to the underlying bone, thus allowing smooth joint motion. The function of articular cartilage is inherently related to the structure and composition of its extracellular matrix. Articular cartilage comprises a biphasic matrix and chondrocytes—the sole cellular component of the tissue embedded within the matrix. Articular cartilage solid matrix mainly comprises of collagen fibers, proteoglycan macromolecules, and water.^10^ The size and orientation of collagen fibers in articular cartilage is a function of tissue depth, varying from the articular surface to the subchondral bone, resulting in three distinct zones, namely: superficial zone, middle zone, and deep zone.^11^ The collagen fibers are oriented parallel to the articular surface in superficial zone, exhibit a skewed orientation in middle zone, and have a perpendicular orientation in deep zone.^10^ Furthermore, there is a mineralized layer of calcified cartilage at the interface between cartilage matrix and subchondral bone.^12^ The unique alignment of collagen fibers and the high osmotic pressure created by proteoglycans equip cartilage matrix with unique biomechanical properties to withstand various mechanical stresses.

In the past two decades, various studies have reported the $\mu_{a}$ and $\mu_{s}^{\prime }$ of articular cartilage and assessed their potential for characterizing the tissue.^13^^–^^22^ However, little attention is given to how articular cartilage zonal architecture affects light propagation through the tissue matrix. In particular, the scattering properties of articular cartilage zones and their impact on the overall optical properties and response of the bulk tissue are not well understood. Few studies in the literature have investigated $\mu_{s}$ and g values of articular cartilage bulk tissue and zones.^13^^,^^23^^,^^24^ Beek et al.^13^ reported g of bulk rabbit cartilage tissue at 632.8 nm using a double-integrating sphere and the inverse adding doubling method.^25^ Shyu et al.^23^ reported the effective g of bulk porcine articular cartilage estimated by optical coherence tomography. Jambor et al.^24^ utilized goniometric setup to estimate the $\mu_{s}$ and g of articular cartilage superficial, middle, and deep zones at 445 and 890 nm and showed how enzymatic degradation such as depletion of proteoglycan and disruption of the collagen mesh will affects these parameters.

The present study aims to provide the spectral values of $\mu_{s}$ and g of healthy articular cartilage zones from different anatomical origins within the bovine knee. We hypothesized the unique compositional and structural properties of articular cartilage zones affects the scattering properties of the tissue and this influence is represented in the scattering properties of articular cartilage zones. We carried out a series of optical spectroscopic and imaging measurements to validate this hypothesis.

## 2 Materials and Methods

### 2.1 Sample Preparation

In this study, intact bovine knee joints (n=15) collected from a local abattoir were used; thus, no ethical permission was required. In the collection process, the joints were selected from skeletally mature animals and no cofounding factors, such as age and sex, were controlled. The joints were obtained within 1 week of slaughter and kept at 4°C before sample extraction. Two sets of adjacent osteochondral samples were harvested from the lateral and medial sides of the femur, tibia, and patella of the joints. Samples in both sets (sets A and B) were extracted using a diameter punch (d=15  mm). After the osteochondral plug extraction, the bone end of each sample was filed until it was parallel to its surface. During the process, the cartilage surface of the plugs was continuously rinsed with phosphate-buffered saline solution (PBS, pH 7.4, containing inhibitors) to minimize deterioration, such as interstitial water evaporation.

Subsequently, the cartilage layer of samples in set A was extracted and subjected to integrating sphere measurement to determine the broadband $\mu_{a}$ and $\mu_{s}^{\prime }$ and polarized light microscopy imaging to estimate the collagen fiber orientation. For samples in set B, cartilage sections (thickness=100  μm) were extracted from the different depth-wise zones and subjected to collimated transmittance measurements to estimate their broadband $\mu_{s}$. Table 1 describes the number of samples collected in each set and their anatomical origin.

**Table 1:** Details of the samples collected in sets A and B. FL, lateral femoral group; FM, medial femur group; PL, lateral patella group; PM, medial patella group; TL, lateral tibia plateau group; and TM, medial tibia plateau. SZ, superficial zone; MZ, medial zone; and DZ, deep zone of articular cartilage, respectively. IS, integrating sphere measurements; PLM, polarized light microscopy imaging; and CT, collimated transmittance measurements.

Measurement type	Sample set		Anatomical origin						Total No.
			FL	FM	PL	PM	TL	TM	
IS	Set A		14	14	12	11	9	8	68
PLM	Set A		6	9	2	3	4	3	27
CT	Set B	SZ	4	4	5	2	4	2	21
		MZ	5	6	6	2	5	4	28
		DZ	6	7	7	2	6	3	31

### 2.2 microCT Imaging

To estimate the thickness and surface diameter of the articular cartilage samples, set A was subjected to microCT imaging immediately after extraction. The samples were imaged with a microCT scanner (XT H 225, Nikon Metrology, Leuven, Belgium). The images were acquired with $40\times 40\times 40\;\mu m^{3}$ isotropic voxel size, but the voxel size was increased to $50\times 50\times 50\;\mu m^{3}$ when reconstructions were calculated. The tube voltage was set to 80 kVp with a 1.0 mm aluminum filter.

**Fig. 1:**
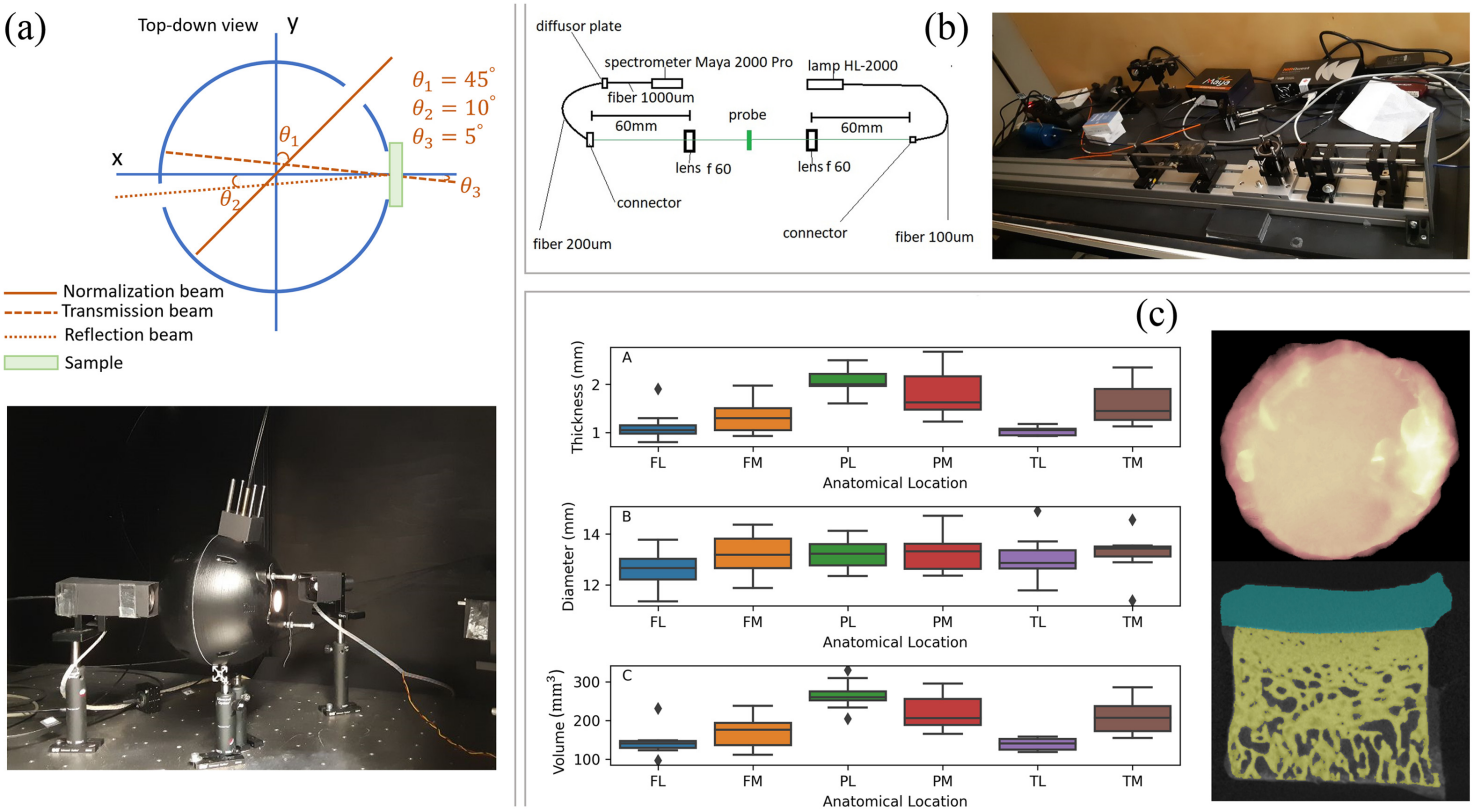
(a) The schematics and image of the integrating sphere setup used for estimation of $\mu_{a}$ and $\mu_{s}^{\prime }$ of articular cartilage bulk tissue. (b) The schematic and image of the collimated transmittance setup used for estimation of $\mu_{t}$ of articular cartilage zones. (c) The distribution of the physical properties (thickness (mm), surface diameter (mm), and volume (${\mathrm{mm}}^{3}$) of the articular cartilage bulk samples, estimated from the microCT images. (c) (right) The top surface of an articular cartilage sample after segmentation and the X-Z cross-section of the sample, which is segmented to articular cartilage (cyan), bone (yellow), and background medium (black). The error of the articular cartilage volume segmentation from the microCT images was ∼<18%.

After image acquisition and reconstruction, the thickness and surface diameter of the articular cartilage part of the osteochondral samples were estimated via processing the microCT images. In summary, the 3D osteochondral microCT images were segmented to obtain the volume of the articular cartilage segment. Considering that the microCT image histogram has three peaks, it can be readily segmented into three classes using histogram thresholding. The classes are bone, soft tissue (articular cartilage and moisture), and background (including air and the sample holder). Distinguishing moisture from articular cartilage is not possible using their intensity values alone. Therefore, the segmentation was done using morphological operators and surface normal vectors to geometrically isolate the articular cartilage tissue from the moisture.^26^ Figure 1 depicts the processing of the microCT images of the osteochondral samples. After microCT imaging, the articular cartilage portion of the osteochondral samples was mechanically detached from sunchondral bone using a scalpel, and then samples were stored in PBS at −20.

### 2.3 Cartilage Cryosection Procedure

Samples in set B were further processed to extract tissue sections from the different cartilage zones. The bone end of each sample was mounted on a disc using a fixative solution (Optimal Cutting Temperature compound, Thermo Fisher Scientific Ltd., Runcorn, United Kingdom). The assembly was placed in a cryostat chamber (Leica CM3050 S, Leica Biosystems, Wetzlar, Germany) for 5 min at −18 to allow sample freezing. Afterward, the assembly was placed on the rotary microtome within the cryostat chamber. Before sectioning, the first 50  μm of the articular surface was cut and discarded to provide a uniformly smooth and flat surface for sectioning. A series of sections with a thickness of 100  μm was then consequently obtained. The sections were hydrated with PBS and sandwiched between a glass slide (thickness = 1 mm, Menzel-Gläser Frosted Microscope Slides, ThermoFisher Scientific, Oy, Finland) and a coverslip (thickness = 0.13 mm, Menzel Microscope Coverslips, ThermoFisher Scientific, Oy, Finland) and then stored in a humid box at −20 for collimated transmittance measurement. The selection of the sections to present the zonal structure of articular cartilage was carried out as follows: (I) the first section was always considered superficial zone, (II) the subsequent section was considered to represent middle zone, and (III) the deep zone layer was selected after the first 500  μm of the tissue.

### 2.4 Integrating Sphere Measurements

The optical setup used in this study comprises an optical apparatus to measure the optical response of biological tissues (reflectance and transmittance) and an optical simulation software to estimate the optical properties ($\mu_{a}$ and $\mu_{s}^{\prime }$) from the optical response of tissues. The optical apparatus is an optimized double-beam integrating sphere setup consisting of a halogen light source (Halostar Starlite, OSRAM, Germany), a visible-band spectrometer with 3-nm resolution (Maya2000Pro, Ocean Optics, USA), a near-infrared (NIR)-band spectrometer with 7-nm resolution (NIRQuest512-1.7, Ocean Optics, United States), an in-house 3D-printed integrating sphere, and a glass sample holder construct [Fig. 1(a)]. Technical details of the optical setup, including its calibration and validation of the measured optical properties, are reported elsewhere.^22^^,^^26^^,^^27^

To estimate the optical properties of articular cartilage, the spectral band of 400 to 1400 nm was used as $\mu_{a}$ of water is low and allows the detection of the spectral features of other chromophores in articular cartilage. In addition, given the higher values of $\mu_{s}^{\prime }$ in this band and high values of g, a higher penetration depth can be achieved that facilitates the extraction of biologically relevant features from deep within the tissue. Moreover, in the absence of spectral values of n and g for bulk articular cartilage, fixed values of 1.358 and 0.9 from the literature were used for n and g.^28^^,^^29^ The in-house optical simulation software was used to implement a 3D Monte Carlo Solver and Henyey–Greenstein scattering phase function^26^ to simulate the angular distribution of scattered photons. The optical simulation software considered the samples to be perfect cylinders. Hence, the lateral radius and axial thickness of articular cartilage samples from Set A, estimated using microCT imaging, were applied to build the cylindrical geometry of the sample in the software.

Before optical measurement, the samples from set A were thawed to room temperature for 30 min. Subsequently, they were placed within a cylindrical sample holder of glass cuvettes to reduce the refractive index mismatch between the tissue and the surrounding medium [Fig. 1(a)]. To estimate the values of $\mu_{a}$ and $\mu_{s}^{\prime }$, we hypothesized that during the integrating sphere measurements, the surrounding medium, filling the gap between articular cartilage and the inner walls of the sample holder, is air to account for any potential morphological irregularities of the samples.

### 2.5 Collimated Transmittance Measurements

The articular cartilage sections extracted from set B samples were subjected to collimated transmittance measurements. The optical setup consists of (I) a halogen lamp (HL-2000, Ocean Insight, United States) as the light source; (II) a spectrometer (Maya 2000 Pro, Ocean Insight, United States); (III) two lenses with regular achromats of f-60 mm and a diameter of 25.4 mm (LINOS Photonics Inc., United States); (IV) an illumination fiber with 100-μm core diameter; (V) detection fiber with a 200-μm core diameter; (VI) a diffusor disc; and (VII) an optical fiber with 1000-μm core diameter, connecting the diffusor disc to the spectrometer. The collimated transmittance measurements were carried out in the spectral band of 400 to 1400 nm. The optical resolution of the used spectrometer in the spectral, with an entrance slit of 100  μm, is around 3 nm. Figure 1(b) shows the schematics and images of the collimated transmittance setup.

The extinvtion coefficient, $\mu_{t}({\mathrm{mm}}^{-1})$, of set B samples were estimated by applying Beer–Lambert’s Law^3^ on the collimated transmittance dataset. In particular, $\mu_{t}$ was obtained as

$$\mu_{t}=-\frac{\log(\frac{T}{T_{0}})}{d},$$ (1)

where $T(=T_{\text{sample}}-T_{\text{Dark}})$ is the transmittance signal of the samples subtracted by the dark current noise of the system ($T_{\text{Dark}}$) and normalized by the detector dynamic range ($T_{0}=T_{\text{Reference}}-T_{\text{Dark}}$). $T_{\text{Reference}}$ is the transmittance measurement when no sample is placed in the system. Finally, d(mm) is the thickness of the samples. Table 1 depicts the number of samples per anatomical location and zonal layer for collimated transmittance measurements.

### 2.6 Polarized Light Microscopy

This study subjected the set A samples to histological processing to prepare thin axial tissue sections for acquiring information about the orientation of collagen fibers in articular cartilage using polarized light microscopy imaging.^30^ After the integrating sphere measurements, the specimens were placed into a fixative solution containing formaldehyde (4%, Merck, Darmstadt, Germany) and ethylenediaminetetraacetic acid (EDTA, 10%, Merck, Darmstadt, Germany), and were kept at room temperature for 21 days. Following fixation, the process continued by dehydrating the specimens with a series of graded ethanol-based solutions and embedding the dehydrated specimens in paraffin for sectioning. The embedded specimens were halved from sagittal plane, and one set of three unstained deparaffinized 5-μm axial sections was obtained from each half.

Fixed tissue sections obtained from set A samples were subjected to polarized light microscopic imaging,^30^^,^^31^ using an apparatus equipped with a microscope body (Leitz Ortholux II POL, Leitz Wetzlar, Germany), a monochromatic light source (λ=630±30  nm, Edmund Optics Inc., Barrington, New Jersey, United States), crossed polarizers (Techspec optics^®^ XP42-200, Edmund Optics, Barrington, New Jersey, United States), and a monochrome camera (pixel size 3.5  μm, BFS-U3-88S6M-C FLIR Blackfly^®^ S, FLIR Systems Inc., United States) with a 2.5× magnification objective. The setup consists of a sample placed between a polarizer and an analyzer at a 90 deg angle. Samples were measured at 21 different orientation angles in the band of (0 deg, 180 deg) with a 9 deg step size.

The collagen network of articular cartilage induces birefringence.^32^ Thus, by measuring the intensity of the polarized light at several angles, a parallelism index can be defined as a measure of collagen fiber orientation using Michelson contrast and the Stokes parameters ($S_{0}$, $S_{1}$, and $S_{2}$). A detailed description of the algorithm for estimating the collagen fiber orientation is reported elsewhere.^31^

Table 1 depicts the number of samples per anatomical location used for polarized light microscopy imaging. Six measurements were carried out for each sample and the results were averaged to provide a depth-wise orientation of collagen fibers for the samples. On average, the superficial zone of the bovine cartilage sample used in this study was 8% of the total thickness, the middle zone was 21% of the total thickness, and the deep zone was 71% of the total thickness.

### 2.7 Estimation of Scattering Parameters

When photons undergo a single scattering event in the tissue under investigation, the Mie–Rayleigh formula empirically describes $\mu_{s}$ of biological tissues as a mixture of photon scattering by the Rayleigh and Mie scatterers, weighted by their normalized contribution fraction. The intensity of Rayleigh-scattered photons varies proportionally to $\lambda^{-4}$, whereas that of Mie-scattered photons varies proportionally to $\lambda^{-b}$, where b is a parameter related to the size of the Mie scatterers and λ is the wavelength of investigation.^3^ Hence, the Mie–Rayleigh formula describes $\mu_{s}$ as

$$\mu_{s}=\alpha [c\times {\left(\frac{\lambda }{\lambda_{0}}\right)}^{-4}+(1-c)\times {\left(\frac{\lambda }{\lambda_{0}}\right)}^{-b}],$$ (2)

where α (= $\mu_{s}(\lambda_{0})$, ${\mathrm{mm}}^{-1}$) is a parameter related to the density of the scatterer particles in the tissue, $\lambda_{0}$ (= 500 nm) is a reference wavelength used for nondimensionalization, and c is the normalized contribution fraction of the Rayleigh scatterers to $\mu_{s}$. Similarly, when photons undergo multiple scattering events in the tissue, $\mu_{s}^{\prime }$ can be described by the Mie–Rayleigh formula, hence

$$\mu_{s}^{\prime }=\alpha^{*}[c^{*}\times {\left(\frac{\lambda }{\lambda_{0}}\right)}^{-4}+(1-c^{*})\times {\left(\frac{\lambda }{\lambda_{0}}\right)}^{-b^{*}}].$$ (3)

$\alpha^{*}$ (= $\mu_{s}^{\prime }(\lambda_{0})$, ${\mathrm{mm}}^{-1}$), $c^{*}$, and $b^{*}$ are the scatterer density parameter, normalized contribution fraction of Rayleigh scatterers to $\mu_{s}^{\prime }$, and the size parameter of Mie scatterers, respectively.

For both cases of $\mu_{s}$ and $\mu_{s}^{\prime }$, the Mie–Rayleigh parameters are estimated by fitting the associated Mie–Rayleigh equations to the experimental values of $\mu_{t}$ and $\mu_{s}^{\prime }$, respectively. As articular cartilage has low $\mu_{a}$, thus $\mu_{t}$ is assumed to be mainly determined by $\mu_{s}$ ($\mu_{t}\approx \mu_{s}$). After removing outliers (n=2), the mean $R^{2}$ and RMSE scores of Mie–Rayleigh fit for $\mu_{s}$ are 96.84% and 1.1576. The mean $R^{2}$ and RMSE scores of Mie–Rayleigh fit for $\mu_{s}^{\prime }$ are 98.83% and 0.0909.

The scattering anisotropy factor (g) is defined as

$$g=1-\frac{\mu_{s}^{\prime }}{\mu_{s}}.$$ (4)

Note that g is dependent on the scattering phase function used for estimating $\mu_{s}^{\prime }$. Given that the Henyey–Greenstein scattering phase function is used in this study to estimate $\mu_{s}^{\prime }$, the estimated broadband values of g are representative of Mie type of scatterers that are best described by the Henyey–Greenstein phase function ($g=g_{\mathrm{HG}}$). Nevertheless, the estimated values of $g_{\mathrm{HG}}$ can be transferred to another set of values ($g_{m\mathrm{HG}}$) that can account for the contribution of Rayleigh scatterers to the photon scattering in articular cartilage as follows:

$$g_{m\mathrm{HG}}=g_{\mathrm{HG}}(1-c).$$ (5)

Where c is the normalized volume fraction of the Rayleigh scatterers. Equation (5), proposed by Graaff et al.,^33^ estimates the impact of Rayleigh scatterers on g of biological tissues.

Similar to the Mie–Rayleigh formula, the Mie-collagen formula describes the scattering properties of biological tissues as scattering due to Mie scatterers and small cylindrical particles with nm-scale diameter. The Mie-collagen power law for $\mu_{s}$ and $\mu_{s}^{\prime }$ can be described as

$$\mu_{s}=\alpha^{**}[c^{**}\times {\left(\frac{\lambda }{\lambda_{0}}\right)}^{-3}+(1-c^{**})\times {\left(\frac{\lambda }{\lambda_{0}}\right)}^{-b^{**}}],$$ (6)

$$\mu_{s}^{\prime }=\alpha^{***}[c^{***}\times {\left(\frac{\lambda }{\lambda_{0}}\right)}^{-3}+(1-c^{***})\times {\left(\frac{\lambda }{\lambda_{0}}\right)}^{-b^{***}}],$$ (7)

where $\alpha^{**}$ (= $\mu_{s}(\lambda_{0})$, ${\mathrm{mm}}^{-1}$) and $\alpha^{***}$ (= $\mu_{s}^{\prime }(\lambda_{0})$, ${\mathrm{mm}}^{-1}$) are the scatterer density parameters; $b^{**}$ and $b^{***}$ are the Mie scatterer size parameters; and $c^{**}$ and $c^{***}$ are the contribution fraction of collagen scatterers to $\mu_{s}$ and $\mu_{s}^{\prime }$, respectively.^22^ This empirical formula was suggested by Kienle et al.,^22^ as they observed through their research that the three-order power function fits the scattering coefficients better than the four-order power function (Rayleigh scattering) for biological tissues that are predominantly composed of collagen fibers. The results of data analysis on Mie-collagen scattering formula are presented in the Supplementary Material. After removing outliers (n=2), the mean $R^{2}$ and RMSE scores of Mie-collagen fit for $\mu_{s}$ are 96.8% and 1.1598. The mean $R^{2}$ and RMSE scores of Mie-collagen fit for $\mu_{s}^{\prime }$ are 98.84% and 0.0906.

### 2.8 Statistical Analysis

Statistical tests were conducted to investigate if the difference in the Mie–Rayleigh parameters per articular cartilage zone and anatomical location are significant.

1. Test 1: statistical difference of Mie–Rayleigh parameters between superficial, middle, and deep zones, and bulk articular cartilage across all anatomical locations.
2. Test 2: statistical difference of Mie–Rayleigh parameters betweem superficial, middle, and deep zones and bulk tarticular cartilage within each anatomical location.
3. Test 3: statistical difference of Mie–Rayleigh parameters between samples obtained from different anatomical locations across all articular cartilage zones.
4. Test 4: statistical difference of Mie–Rayleigh parameters between samples obtained from different anatomical locations per articular cartilage zone.

The statistical test comprises the Kolmogorov–Smirnov normality test, Leven’s test for equality of variances, one-way ANOVA and Tukey’s tests to examine the difference in normally-distributed observations, and Kruskal-Wallis and Dunn’s tests for nonparameteric observations. All the computational and statistical analyses required for the present report were carried out in MATLAB (R2019b and R2020b) and Python v3.7 using standard libraries.

## 3 Results

**Fig. 2:**
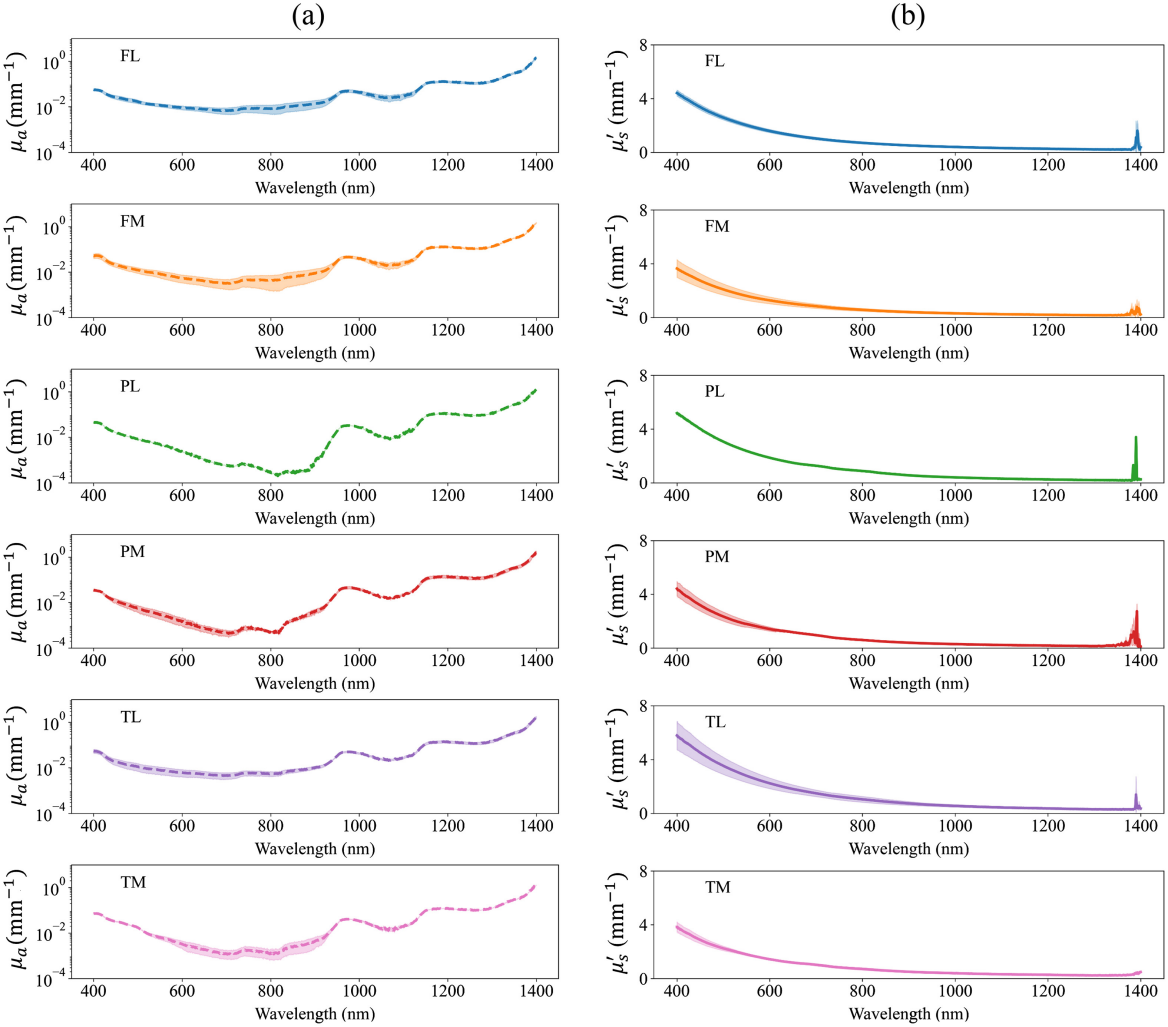
Absorption coefficient (a) ($\mu_{a}$, ${\mathrm{mm}}^{-1}$) and reduced scattering coefficient (b) ($\mu_{s}^{\prime }$, ${\mathrm{mm}}^{-1}$) of bulk articular cartilage tissue. FL and FM are the lateral and medial femur sites, PL and PM are the lateral and medial patella sites, and TL and TM are the lateral and medial tibia sites. The values represent mean ± standard deviation.

**Fig. 3:**
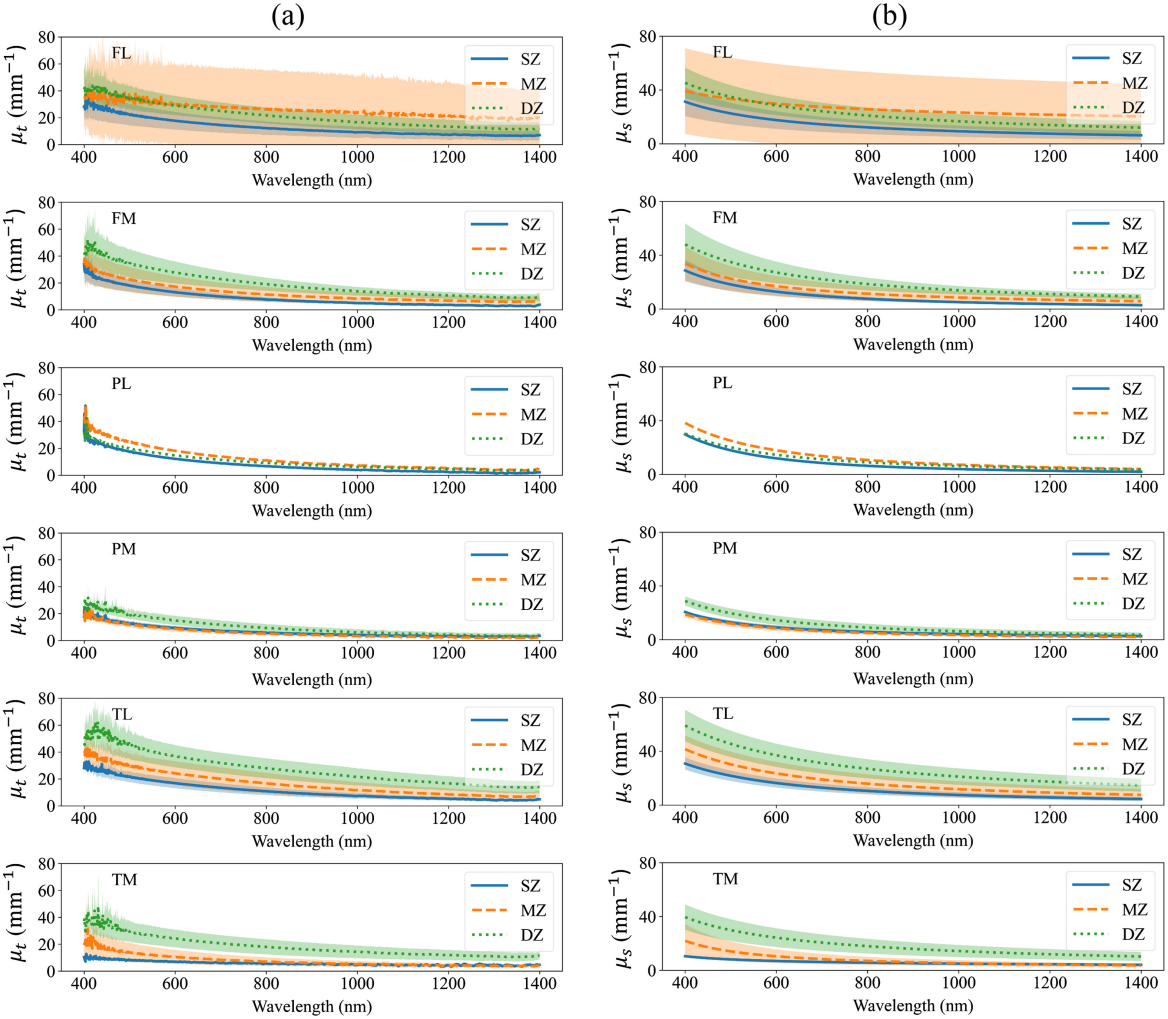
The extinction coefficient (a) ($\mu_{t}$, ${\mathrm{mm}}^{-1}$) and scattering coefficient (b) ($\mu_{s}$, ${\mathrm{mm}}^{-1}$) of articular cartilage zones of different anatomical locations. $\mu_{t}$ was experimentally obtained from collimated transmittance measurements while $\mu_{s}$ was obtained by fitting the Mie–Rayleigh formula to the experimental $\mu_{t}$. SZ, MZ, and DZ are the superficial, middle, and deep zones of articular cartilage, respectively. FL and FM are the lateral and medial femur sites, PL and PM are the lateral and medial patella sites, and TL and TM are the lateral and medial tibia sites. The values represent mean ± standard deviation.

The values of $\mu_{a}$ of bulk articular cartilage suggest that the tissue possesses low light absorption strength with apparent water absorption peaks at 950 and 1150 nm [Fig. 2(a)]. On average, the decrease of values of bulk articular cartilage $\mu_{s}^{\prime }$ shows the presence of Rayleigh scatterers in the tissue and their contribution to $\mu_{s}^{\prime }$ [Fig. 2(b)]. The similarity of bulk articular cartilage $\mu_{a}$ and $\mu_{s}^{\prime }$ over different anatomical locations suggest anatomical variation has minimal impact on the bulk optical properties [Figs. 2(a) and 2(b)]. The values of $\mu_{t}$ of articular cartilage zones are predominantly influenced by the tissue’s $\mu_{s}$, with minimal impact of $\mu_{a}$, including the water absorption peaks at 950 and 1150 nm (Fig. 3). Mean values of $\mu_{s}$ and $\mu_{t}$ exhibited higher values in deep zone than middle zone and superficial zones over the anatomical sites of medial femur and patella, and lateral and medial tibia. In addition, similar to $\mu_{s}^{\prime }$, the decrease of $\mu_{s}$ of articular cartilage zones suggests the presence of Rayleigh scattering and its contribution to $\mu_{s}$ [Fig. 3(b)].

**Fig. 4:**
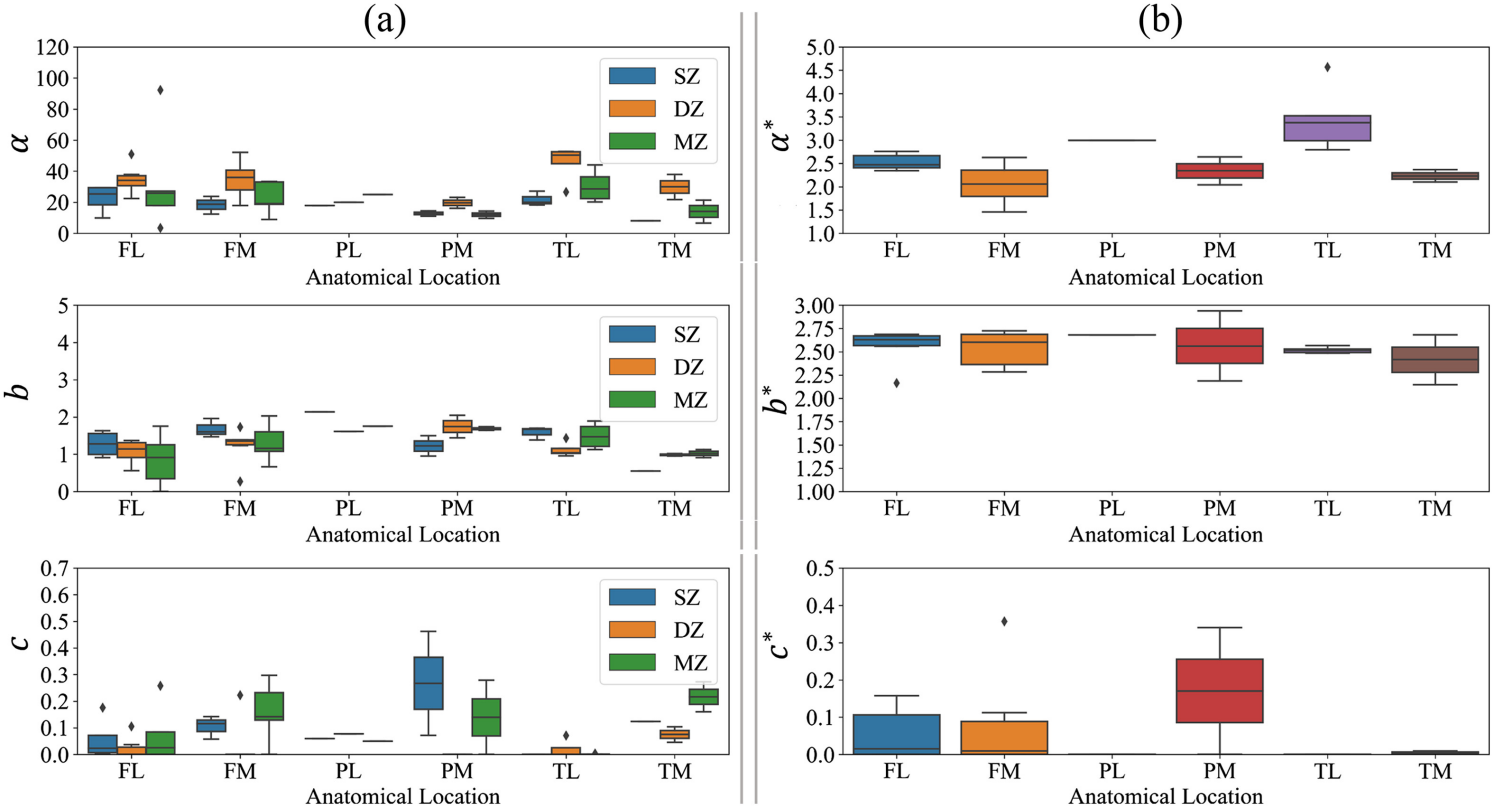
(a) The Mie–Rayleigh parameters obtained from fitting the $\mu_{s}$ of articular cartilage zones to Mie–Rayleigh formula over different anatomical location. (b) The Mie–Rayleigh parameters obtained from fitting the $\mu_{s}^{\prime }$ of bulk articular cartilage tissue to Mie–Rayleigh formula over different anatomical location. SZ, MZ, and DZ are the superficial, middle, and deep zones of articular cartilage, respectively. FL and FM are the lateral and medial femur sites, PL and PM are the lateral and medial patella sites, and TL and TM are the lateral and medial tibia sites. α and $\alpha^{*}$ are the scatterer-density parameter of $\mu_{s}$ and $\mu_{s}^{\prime }$; b and $b^{*}$ are the scatterer-size parameter of $\mu_{s}$ and $\mu_{s}^{\prime }$; and c and $c^{*}$ are the normalized contribution of Rayleigh scatterers to $\mu_{s}$ and $\mu_{s}^{\prime }$, respectively.

When the Mie–Rayleigh parameters of $\mu_{s}$ of cartilage zones are considered [Fig. 4(a)], on average and across all anatomical locations, the scatterer density parameter (α) increases consistently with tissue depth. That is, α is highest in deep zone and also higher in middle zone than in superficial zone (Table 2). The scatterer size parameter (b) shows a similar trend to α when averaged across all anatomical locations. However, the contribution of Rayleigh scatterer to $\mu_{s}$ (c) is highest in middle zone with a mean value of 10.65% across all anatomical locations (Table 2). When the Mie–Rayleigh parameters of bulk articular cartilage $\mu_{s}^{\prime }$ are considered [Fig. 4(b)], on average, the scatterer density parameter ($\alpha^{*}$) is higher in the lateral groups (lateral femur, tibia, and patella) than in the medial groups (medial femur, tibia, and patella). The scatterer size parameter ($b^{*}$) shows a similar trend in the lateral and medial groups. However, when the contribution of Rayleigh scatterer to $\mu_{s}^{\prime }$ ($c^{*}$) is considered, the medial groups possess larger values of $c^{*}$ than the lateral groups (Table 2).

**Table 2:** The mean values of the scatterer-density parameter (α and $\alpha^{*}$), the scatterer-diameter parameter (b and $b^{*}$) and normalized contribution of Rayleigh scatterers (c and $c^{*}$) to the scattering coefficients of articular cartilage zones and bulk tissue across different anatomical location. $\mu_{s}$ and $\mu_{s}^{\prime }$ are the single and reduced scattering coefficients. SZ, MZ, and DZ are the superficial, middle, and deep zones of articular cartilage, respectively. FL and FM are the lateral and medial femur sites; PL and PM are the lateral and medial patella sites; and TL and TM are the lateral and medial tibia sites. The Mie–Rayleigh parameters were obtained by fitting Eqs. (2) and (3) to $\mu_{s}$ and $\mu_{s}^{\prime }$ values, respectively. The mean $R^{2}$ and RMSE scores of Mie–Rayleigh fit for $\mu_{s}$ are 96.84% and 1.1576. The mean $R^{2}$ and RMSE scores of Mie–Rayleigh fit for $\mu_{s}^{\prime }$ are 98.83% and 0.0909.

Mie–Rayleigh parameter	Zonal structure	Anatomical location					
		FL	FM	PL	PM	TL	TM
α ($\mu_{s}$)	SZ	22.5 ± 7.99	18.23 ± 4.70	17.84 ± 0.0	12.75 ± 1.63	21.7 ± 3.86	8.08 ± 0.0
	MZ	33.25 ± 30.64	22.60 ± 9.36	25.02 ± 0.0	11.95 ± 2.29	30.23 ± 9.41	14.08 ± 7.38
	DZ	34.83 ± 8.66	34.94 ± 11.02	20.05 ± 0.0	19.64 ± 3.56	45.44 ± 9.82	29.86 ± 8.03
$\alpha^{*}$ ($\mu_{s}^{\prime }$)	Bulk	2.53 ± 0.16	2.06 ± 0.4	3.0 ± 0.0	2.34 ± 0.3	3.45 ± 0.62	2.23 ± 0.13
b ($\mu_{s}$)	SZ	1.27 ± 0.31	1.68 ± 0.2	2.14 ± 0.0	1.22 ± 0.27	1.58 ± 0.14	0.55 ± 0.0
	MZ	0.85 ± 0.63	1.30 ± 0.47	1.75 ± 0.0	1.69 ± 0.05	1.49 ± 0.31	1.02 ± 0.11
	DZ	1.07 ± 0.29	1.22 ± 0.45	1.61 ± 0.0	1.74 ± 0.30	1.12 ± 0.17	0.98 ± 0.03
$b^{*}$ ($\mu_{s}^{\prime }$)	Bulk	2.56 ± 0.18	2.53 ± 0.18	2.68 ± 0.0	2.56 ± 0.37	2.52 ± 0.03	2.41 ± 0.27
c ($\mu_{s}$)	SZ	0.05 ± 0.07	0.10 ± 0.03	0.06 ± 0.0	0.27 ± 0.19	0.0 ± 0.0	0.12 ± 0.0
	MZ	0.07 ± 0.1	0.16 ± 0.1	0.05 ± 0.0	0.14 ± 0.14	0.0 ± 0.0	0.22 ± 0.06
	DZ	0.02 ± 0.04	0.04 ± 0.08	0.08 ± 0.0	0.0 ± 0.0	0.02 ± 0.03	0.07 ± 0.03
$c^{*}$ ($\mu_{s}^{\prime }$)	Bulk	0.05 ± 0.06	0.08 ± 0.13	0.0 ± 0.0	0.17 ± 0.17	0.0 ± 0.0	0.0 ± 0.0

The results of the statistical analysis indicate a nonuniform statistically significant difference between the Mie–Rayleigh parameters of $\mu_{s}$ of the articular cartilage zones and anatomical sites (Table 3). In particular, α is significantly different between superficial and deep zones (p-value=0.001) and between middle and deep zones (p-value=0.015). However, b and c are not statistically significant across the different zones (Table 3).

**Table 3:** The p-value of posthoc method of group statistical test for assessing the statistically significant difference of Mie–Rayleigh parameters of $\mu_{s}$ over different articular cartilage zones and anatomical locations. $\mu_{s}$ is the single scattering coefficient of articular cartilage tissue. α is the scatterer-density parameter, b is the scatterer-size parameter, and c is the contribution of the Rayleigh scatterers to $\mu_{s}$ of articular cartilage zones. SZ, MZ, and DZ are the superficial, middle, and deep zones of articular cartilage, respectively. FL and FM are the lateral and medial femur sites, PL and PM are the lateral and medial patella sites, and TL and TM are the lateral and medial tibia sites. The italicized values highlight the statistical significance difference.

Optical property	Mie–Rayleigh parameter	Zone	Zone					
			SZ		MZ		DZ	
$\mu_{s}$	α	SZ	1.0		0.9694		*0.0010*	
		MZ	0.9694		1.0		*0.015*	
		DZ	*0.001*		*0.015*		1.0	
	b	SZ	1.0		1.0		0.3655	
		MZ	1.0		1.0		1.0	
		DZ	0.3655		1.0		1.0	
	c	SZ	1.0		1.0		0.1082	
		MZ	1.0		1.0		0.1328	
		DZ	0.1082		0.1328		1.0	
	Mie–Rayleigh parameter	Anatomical location	Anatomical location					
			FL	FM	PL	PM	TL	TM
	α	FL	1.0	1.0	1.0	*0.0003*	*0.0009*	0.6379
		FM	1.0	1.0	1.0	*0.00003*	*0.0001*	0.4552
		PL	1.0	1.0	1.0	*0.0*	*0.0*	0.0687
		PM	*0.0003*	*0.00003*	*0.0*	1.0	1.0	1.0
		TL	*0.0009*	*0.0001*	*0.0*	1.0	1.0	1.0
		TM	0.6379	0.4552	0.0687	1.0	1.0	1.0
	b	FL	1.0	1.0	1.0	*0.0003*	*0.0009*	0.6379
		FM	1.0	1.0	1.0	*0.00003*	*0.0001*	0.4552
		PL	1.0	1.0	1.0	*0.0*	*0.0*	0.0687
		PM	*0.0003*	*0.00003*	*0.0*	1.0	1.0	1.0
		TL	*0.0009*	*0.0001*	*0.0*	1.0	1.0	1.0
		TM	0.6379	0.4552	0.0687	1.0	1.0	1.0
	c	FL	1.0	1.0	1.0	*0.0003*	*0.0009*	0.6379
		FM	1.0	1.0	1.0	*0.00003*	*0.0001*	0.4552
		PL	1.0	1.0	1.0	*0.0*	*0.0*	0.0687
		PM	*0.0003*	*0.00003*	*0.0*	1.0	1.0	1.0
		TL	*0.0009*	*0.0001*	*0.0*	1.0	1.0	1.0
		TM	0.6379	0.4552	0.0687	1.0	1.0	1.0

In addition, the statistical analysis suggests significant difference of Mie–Rayleigh parameters of articular cartilage $\mu_{s}$ (from all zones) when considered over anatomical locations. α, b, and c are significantly different between lateral femur and medial patella, between lateral femur and tibia, between medial femur and patella, between medial femur and lateral tibia, between lateral and medial patella, and between lateral patella and tibia, all with p-values <0.001 (Table 3). No statistically significant difference were observed for the Mie–Rayleigh scattering parameters of $\mu_{s}^{\prime }$ over different anatomical locations. Furthermore, similar statistical analysis was carried out for the Mie-collagen parameters of $\mu_{s}$ and $\mu_{s}^{\prime }$. The results are presented in the Supplementary Material.

**Fig. 5:**
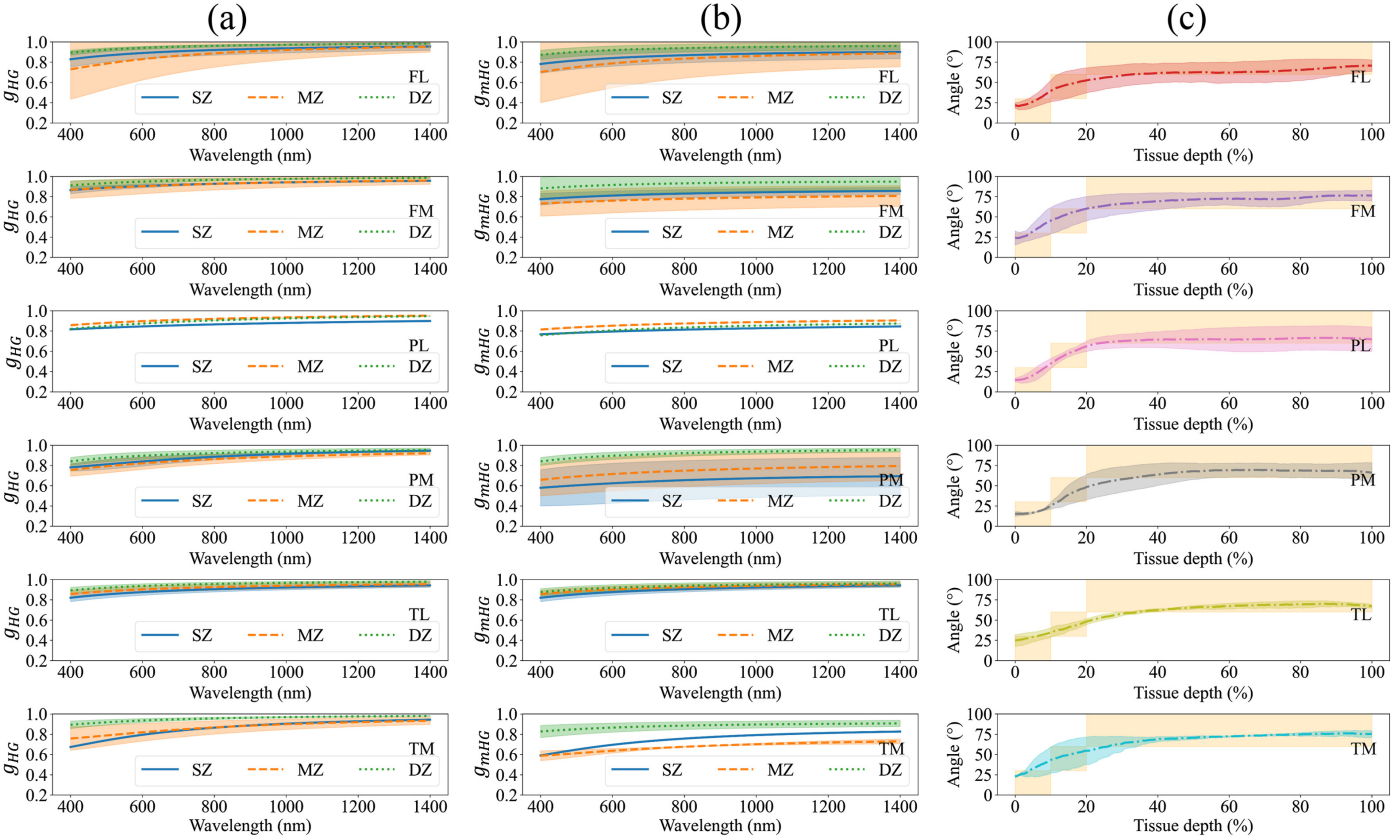
The Henyey–Greenstein scattering anisotropy factor ($g_{\mathrm{HG}}$) of articular cartilage zones (SZ, MZ, and DZ) for different anatomical origins (a). The modified Henyey-Greenstein scattering anisotropy factor ($g_{m\mathrm{HG}}$) of articular cartilage zones (SZ, MZ, and DZ), accounted for the impact of the Rayleigh scatterers on $g_{\mathrm{HG}}$ (b). The depth-wise alignment of collagen fiber of articular cartilage of different anatomical origins (c); the orange shaded areas depicts the percentage of the articular cartilage zones of the total thickness according to the alignment of collagen fibers. SZ, MZ, and DZ are the superficial, middle, and deep zones of articular cartilage defined as the regions where the collagen fibers have alignment of 0 deg to 30 deg, 30 deg to 60 deg, and 60 deg to 90 deg, respectively. FL and FM are the lateral and medial femur sites, PL and PM are the lateral and medial patella sites, and TL and TM are the lateral and medial tibia sites. $g_{\mathrm{HG}}$ and $g_{m\mathrm{HG}}$ are unitless parameters. The values were plotted as mean ± standard deviation.

As observed in Fig. 5(c), deep zone forms a significant part of articular cartilage (∼80%) with collagen angular alignment of ≥60  deg. Whereas superficial (collagen angular alignment of ≤30  deg) and middle zone (30  deg≤ collagen angular alignment of ≤60  deg) contribute roughly to 20% of articular cartilage tissue depth across different anatomical locations. The values of g ($g_{\mathrm{HG}}$ and $g_{m\mathrm{HG}}$) for the different zones [Figs. 5(a) and 5(b)] suggest that light in the visible and NIR bands follows a forward-direction propagation in articular cartilage zones with higher $g_{\mathrm{HG}}$ and $g_{m\mathrm{HG}}$ in the spectral band of 800 to 1400 nm than the short visible band (400 to 800 nm). In addition, across all anatomical locations, $g_{\mathrm{HG}}$ shows a monotonically increasing trend as function of tissue depth. That is $g_{\mathrm{HG}}$ is highest in deep zone; middle zone exhibits the lowest $g_{\mathrm{HG}}$ values across the anatomical sites lateral femur, and medial femur, patella, and tibia, whereas superficial zone possess the lowest $g_{\mathrm{HG}}$ values across the lateral patella and tibia. When the contribution of Rayleigh scatterers are considered, superficial zone has the lowest $g_{m\mathrm{HG}}$ values over the anatomical locations of lateral patella, medial patella, and lateral tibia. Whereas middle zone exhibits lowest $g_{m\mathrm{HG}}$ values over the anatomical locations lateral femur, medial femur, and medial tibia.

## 4 Discussion

This study addresses the gap in knowledge on the scattering properties of articular cartilage depth-wise zones by determining its values of $\mu_{s}$ and g in the different zones, in addition to the depth-wise Mie–Rayleigh parameters. The present findings enhance understanding on the scattering properties of healthy bulk articular cartilage and its depthwise zones. In addition, the depth-wise angular distribution of articular cartilage collagen fibers were provided to investigate the impact of collagen orientation on articular cartilage g. In this study, we hypothesized that normal variation in articular cartilage zones will influence its scattering properties. Our results indicate that α, the Mie-Rayleigh parameter representing scatterer density in articular cartilage, exhibits statistically significant variations throughout the zones of articular cartilage. Furthermore, $g_{\mathrm{HG}}$ and $g_{m\mathrm{HG}}$ of articular cartilage zones show that they vary across the tissue depth.

In our view, various factors affect the values of articular cartilage g, including the depth-wise normalized fraction of the Rayleigh scatterers contribution to the scattering coefficients, the intensity of light scattered by the Rayleigh and Mie scatterers as a function of wavelength, and the heterogeneity of the tissue’s structure. When the contribution of Rayleigh scatterers to $\mu_{s}^{\prime }$ of bulk articular cartilage tissue is considered, on average, ∼5.14% of $\mu_{s}^{\prime }$ can be attributed to these scatterers. Hence, the impact of Rayleigh scatterers on bulk articular cartilage g is expected to be weak ($g_{\mathrm{HG}}\approx g_{m\mathrm{HG}}$). When the contribution of Rayleigh scatterers to $\mu_{s}$ of the different cartilage zones is considered, their impact on g becomes more apparent. On average, these scatterers contribute to 10.19%, 10.65%, and 3.87% of $\mu_{s}$ of articular cartilage superficial, middle, and deep zones, respectively. Therefore, as observed in Fig. 5, the broadband values of articular cartilage g at superficial and middle zones are lower than those at deep zone. When g is considered in the different zones and anatomical locations (Fig. 5), our findings suggest that c (the contribution of the Rayleigh scatterers) acts as a scaling factor on the magnitude of g ($g_{m\mathrm{HG}}\propto cg_{\mathrm{HG}}$), which is supported by Eq. (5).

To understand how articular cartilage g changes as a function of wavelength in the spectral band of 400 to 1400 nm, we investigated this behavior in the different tissue zones, separately from the bulk tissue. This is because collagen orientation within each zone is relatively homogeneous, except in middle zone, but the angular distribution of collagen fibers in the bulk tissue exhibits a wide range of variation, as high as 70 deg [Fig. 5(c)]. Therefore, it is plausible that the spectral behavior of g within articular cartilage zones stems from the ratio between the intensity of light scattered by the Rayleigh and Mie scatterers, whereas in the bulk tissue, the heterogeneity of angular distribution of collagen fibers acts as a confounding factor. As stated earlier, the behavior of g is primarily dependent upon the scattering phase function. The scattering phase function describes the intensity of the scattered light, which is affected by the Rayleigh and Mie scatterers. The intensity of light scattered by the Rayleigh and Mie scatterers varies in proportion to $\lambda^{-4}$ and $\lambda^{-b}$, respectively. On average, the parameter b was estimated as 1.4068, 1.3508, and 1.2925 for articular cartilage superficial, middle, and deep zones, respectively (Table 2). The 4% to 8% difference in b suggests similarities across the zones, which in turn leads to similar trends of $\mu_{s}$ and g (both $g_{\mathrm{HG}}$ and $g_{m\mathrm{HG}}$) in superficial, middle, and deep zones (Figs. 3 and 5).

When the impact of the zonal structure of articular cartilage on g is considered, the importance of the angular distribution of collagen fibers across the tissue depth becomes apparent. 80% of bovine articular cartilage matrix consists of deep zone with collagen fibers oriented between 60 deg and 80 deg [Fig. 5(c)]. Whereas superficial zone (fiber orientation of 0°-30°) and middle zone (fiber orientation of 30 deg to 60 deg) together comprise roughly the first 20% of bovine cartilage matrix. Hence, we speculate that in the short visible spectral band (400 to 700 nm), the structural and scattering features of superficial and middle zones, including collagen orientation and contribution of Rayleigh scatterers to the scattering coefficients, will affect the g values of articular cartilage bulk tissue. This is because of the higher intensity of Rayleigh scattering in this spectral band and the higher contribution of Rayleigh scatterers to $\mu_{s}$ of cartilage superficial and middle zones. In addition, the change of collagen fiber orientation from parallel to the articular surface in superficial zone to a skewed orientation in middle zone is likely to alter the light propagation behavior and cause the values of g to be dependent on the alignment of collagen fibers. This speculation can also be supported by the scattering mean free path ($=1/\mu_{s}$). Due to large values of $\mu_{s}$ in the short visible band, the scattering mean free path decreases while the number of scattering events in superficial and middle zones increases. Hence, the impact of structural and scattering properties of superficial and middle zones on light scattering is higher in this spectral band.

In the far visible and NIR bands (700 to 1400 nm), the values of g for bulk cartilage is affected by the structural and scattering properties of deep zone predominantly. As the photon wavelength shifts from the short visible to the NIR region, the magnitude of $\mu_{s}$ declines and the size of the scattering mean free path increases. Given that articular cartilage is mostly comprised of deep zone with collagen fibers oriented perpendicular to the surface, most of the scattering events occur in a forward direction in deep zone. Hence, bulk articular cartilage is expected to have g values like those of superficial and middle zones in the short visible region and g values close to those of deep zone in the far visible and NIR regions.

Detailed knowledge of the light propagation characteristics in articular cartilage is critical for a better understanding of the origin of the optical response of the tissue. Light penetration depth, depth-origin, and path length of photons propagating the articular cartilage matrix are the key parameters that can shed light on the contribution of the different zones of the articular cartilage on its optical response. This study provides critical input for developing Monte Carlo models of light propagation in articular cartilage to estimate photon penetration depth, depth origin, and path length and subsequently the wavelength-dependent optical response of the tissue.

In this study, certain reasonable assumptions were made. Light propagation in biological tissues are complex. That is, due to complex architecture of their matrix, light propagation becomes anisotropic and tissue scattering coefficients and phase function become dependent on the light’s incident direction. Skin, muscle, and dentin are example of such tissues that exhibit anisotropic light propagation.^33^^,^^34^ We speculate that articular cartilage, due to its zonal architecture, is an example of such biological tissues too. However, we assumed an isotropic light propagation model to investigate the light interaction in articular cartilage and estimate its optical properties. The underlying reason is due to numerous unknowns associated with the anisotropic model of light propagation in articular cartilage that makes its usage not feasible.

In general, the reported $\mu_{s}$ values are from different layers of articular cartilage, whereas the $\mu_{s}^{\prime }$ values are obtained from the bulk of articular cartilage tissue, hence $\mu_{s}^{\prime }$ is a lump-sum representation of the $\mu_{s}^{\prime }$ of articular cartilage layers. However, the low thickness of the tissue’s superficial and middle zones (less than a few hundred micrometers) makes it impossible to extract sufficiently thick sections from superficial and middle zones to estimate their $\mu_{s}^{\prime }$. Therefore, our approach was to estimate the $\mu_{s}^{\prime }$ of bulk articular cartilage and $\mu_{s}$ of articular cartilage zones and investigate the relationship between the scattering properties of the bulk tissue and its zones through [Eq. (4)]. In this context, we assumed $\mu_{s,\text{bulk tissue}}^{\prime }/\mu_{s,\text{cartilage zone}}$ represents a normalized contribution of individual cartilage zones to the scattering properties of bulk tissue.

As the broadband values of n and scattering phase function of bulk articular cartilage tissue are not known and the estimation of $\mu_{a}$ and $\mu_{s}^{\prime }$ depends on these parameters, we used fixed values of n and g (applying to the Henyey–Greenstein scattering phase function used in this study) to estimate its broadband $\mu_{a}$ and $\mu_{s}^{\prime }$ values. Bergmann et al.^22^ reported that the variation of n in the range of 1.3 to 1.5 could result in a relative change of less than 5% for $\mu_{s}^{\prime }$ and <13% for $\mu_{a}$ of biological tissues. More so, in our previous study,^29^ we showed that using fixed value of g (0.8≤g≤0.99) will not result in statistically significant change in the estimation of articular cartilage $\mu_{a}$ and $\mu_{s}^{\prime }$. Thus, we expect that using fixed value of g for estimation of bulk articular cartilage $\mu_{s}^{\prime }$ has minimal impact on the reported values of g for different zones of articular cartilage.

Furthermore, the choice of scattering phase function used in the estimation of $\mu_{s}^{\prime }$ plays a crucial role in determining the spectral values of g, which is inherently dependent upon the scattering phase function, and defined as the first Legendre moment of the scattering phase function.^1^ In this study, the Henyey–Greenstein phase function was used to estimate the $\mu_{s}^{\prime }$ of bulk articular cartilage. As we utilized the relationship between $\mu_{s}^{\prime }$ and $\mu_{s}$ [Eq. (4)] to estimate articular cartilage g, we expect that the estimated values of g will also be dependent upon the Henyey-Greenstein scattering phase function. The Henyey–Greenstein phase function is biased toward the forward scattering of light by Mie scatterers.^1^^,^^3^ Therefore, the impact of the Rayleigh scatterers on the isotropic scattering of light and subsequently g might not be properly captured by the Henyey–Greenstein scattering phase function. To minimize the impact of this bias, Eqs. (2) and (5) were utilized to estimate the volume fraction of the Rayleigh scatterers and to account for their impact on articular cartilage g. We estimated the volume fraction of the Rayleigh scatterers in each zone of articular cartilage from different anatomical origins and showed how the depth-wise normalized contribution of the Rayleigh scatterers to $\mu_{s}$ of articular cartilage will impact the corresponding g values [Table 2 and Fig. 5(b)]. We also provided the spectral values of g based on the Henyey–Greenstein phase function ($g_{\mathrm{HG}}$) and when the contribution of Rayleigh scatterers is accounted for ($g_{m\mathrm{HG}}$). We then enumerated the similarities and differences between $g_{\mathrm{HG}}$ and $g_{m\mathrm{HG}}$ across cartilage zones and various anatomical locations.

Although it is often theoretically expected to observe decreasing broadband values of g with increasing wavelength, our results show an increasing trend in the broadband values of g for articular cartilage. We performed Monte Carlo simulations, for which we considered the actual experimental setup, showing that for our apparatus and $\mu_{t}\leq 50\;{\mathrm{mm}}^{-1}$ and a g≤0.99, there was no significant contribution from multi-scattered light using the Henyey–Greenstein phase function. Hence, the measurement geometry and the resulting values for $\mu_{t}$ should be precise. Therefore, the increasing trend of g observed in this study could be potentially due to the alignment of collagen fibers, a special diameter distributions of the involved collagen fibers, and further scattering structures, such as the chondrocytes. Formulating the impact of these structures in articular cartilage $\mu_{s}$ and its broadband g requires an independent investigation, which is out of the scope of the present study.

## 5 Conclusion

This study reports the broadband $\mu_{s}$ and g of articular cartilage zonal structure in the spectral band of 400 to 1400 nm. To achieve this, we carried out integrating sphere measurements on bulk tissue of articular cartilage and collimated transmittance measurements on thin layers of articular cartilage that represent its zonal structure. Our findings suggest that the depth-wise distribution of the volume fraction of Rayleigh scatterers, the intensity of the light scattered by the Rayleigh and Mie scatterers, and the orientation of collagen fibers across the articular cartilage matrix are the key parameters that affect the broadband values of g of articular cartilage zones and bulk tissue.

## Data Availability

The data reported in this article is available upon reasonable request.
