# Supplementary material for: Broadband scattering properties of articular cartilage zones and their relationship with the heterogenous structure of articular cartilage extracellular matrix
Source: J Biomed Opt. 2023 Dec 13;28(12):125003. doi: 10.1117/1.JBO.28.12.125003 (PMC10718485; doi:10.1117/1.JBO.28.12.125003)
Supplement: Supplementary file 1 [file JBO_028_125003_SD001.pdf]

Supplementary Material

The Mie-collagen power law describes the scattering properties of biological tissues as scattering due to Mie scatterers and small cylindrical particles with nm-scale diameter. The Mie-collagen power law for μ_s and μ_s' can be described as:

$$\mu_s = \alpha^{**}[c^{**} \times \left(\frac{\lambda}{\lambda_0}\right)^{-3} + (1 - c^{**}) \times \left(\frac{\lambda}{\lambda_0}\right)^{-b^{**}}], \quad \text{Eq. (1)}$$

$$\mu_s' = \alpha^{***}[c^{***} \times \left(\frac{\lambda}{\lambda_0}\right)^{-3} + (1 - c^{***}) \times \left(\frac{\lambda}{\lambda_0}\right)^{-b^{***}}], \quad \text{Eq. (2)}$$

where α^{**} (= $\mu_s(\lambda_0)$, mm^{-1}) and α^{***} (= $\mu_s'(\lambda_0)$, mm^{-1}) are the scatterer density parameters, b^{**} and b^{***} are the Mie scatterer size parameters, and c^{**} and c^{***} are the contribution fraction of collagen scatterers to μ_s and μ_s', respectively. The broadband values of μ_s and μ_s' were fitted to Eqs. (1) and (2) to obtain the Mie-collagen parameters. The results are depicted in Fig. 1.

A series of statistical tests were conducted to estimate the statistically significant difference between the Mie-collagen parameters across different zones and anatomical locations. The results of the statistical tests is shown in Table 1. It is noteworthy no statistical difference was for the Mie-collagen parameters obtained from μ_s' when compared across different anatomical locations.

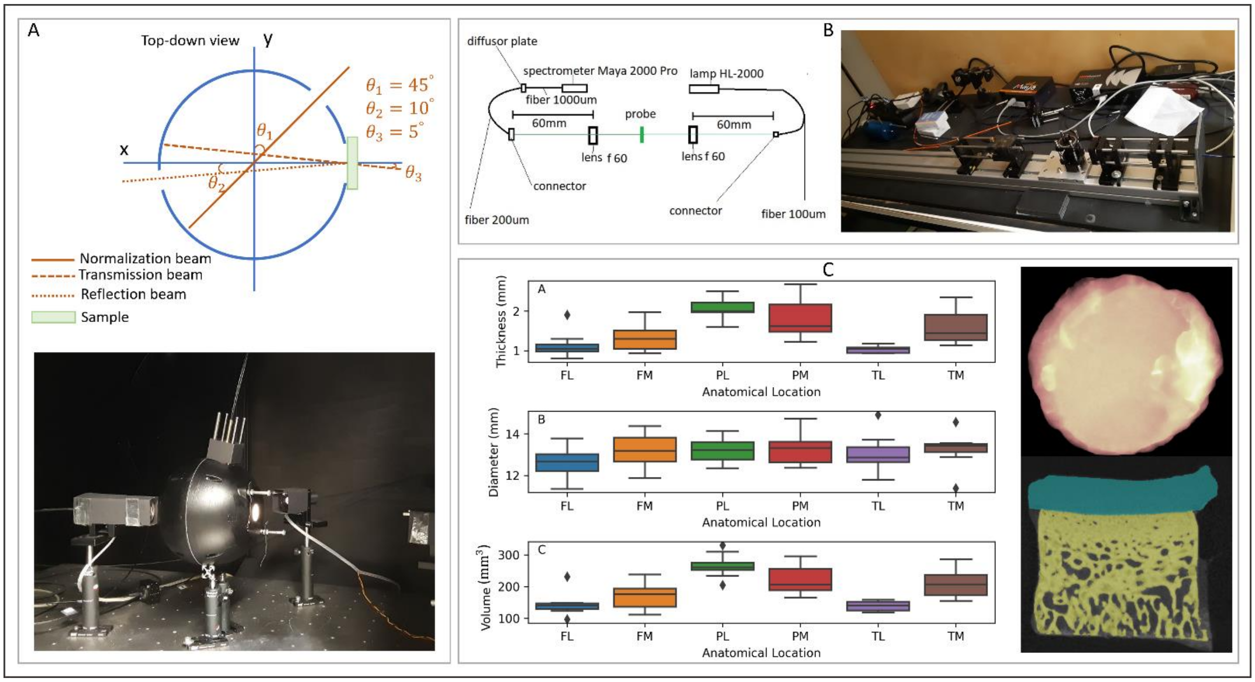

Fig. 1. The Mie-collagen scattering parameters of articular cartilage zones and bulk tissue, obtained from the articular cartilage μ_s and μ_s', respectively. SZ, MZ, and DZ are the superficial, middle, and deep zones of articular cartilage, respectively. FL and FM are the lateral and medial femur sites; PL and PM are the lateral and medial patella sites; and TL and TM are the lateral and medial tibia sites. α^{**} and α^{***} are the scatterer-density parameter of μ_s and μ_s', b^{**} and b^{***} are the Mie scatterer size parameter of μ_s and μ_s', and c^{**} and c^{***} are the normalized contribution of collagen scatterers to μ_s and μ_s', respectively.

Table 1. The *p-value* of posthoc method of group statistical test for assessing the statistically significant difference of Mie-collagen parameters of μ_s over different articular cartilage zones and anatomical locations. μ_s is the single scattering coefficient of articular cartilage tissue. α^{**} is the scatterer-density parameter, b^{**} is the scatterer-size parameter, and c^{**} is the contribution of the Rayleigh scatterers to μ_s of articular cartilage zones. SZ, MZ, and DZ are the superficial, middle, and deep zones of articular cartilage, respectively. FL and FM are the lateral and medial femur sites; PL and PM are the lateral and medial patella sites; and TL and TM are the lateral and medial tibia sites. The tabs highlighted in green color show statistically significant difference.

Optical property	Mie-collagen parameter	Zone	Zone					
			SZ		MZ		DZ	
μ_s	α^{**}	SZ	1.0		0.8711		0.0007	
		MZ	0.8711		1.0		0.0152	
		DZ	0.0007		0.0152		1.0	
	b^{**}	SZ	1.0		0.7546		0.7330	
		MZ	0.7546		1.0		1.0	
		DZ	0.7330		1.0		1.0	
	c^{**}	SZ	1.0		1.0		0.1480	
		MZ	1.0		1.0		0.0485	
		DZ	0.1480		0.0485		1.0	
	Mie-collagen parameter	Anatomical location	Anatomical location					
			FL	FM	PL	PM	TL	TM
	α^{**}	FL	1.0	1.0	1.0	0.0001	0.0005	0.5307
		FM	1.0	1.0	1.0	0.0	0.0002	0.5744
		PL	1.0	1.0	1.0	0.0	0.0	0.0620
		PM	0.0001	0.0	0.0	1.0	1.0	1.0
		TL	0.0005	0.0002	0.0	1.0	1.0	1.0
		TM	0.5307	0.5744	0.0620	1.0	1.0	1.0
	b^{**}	FL	1.0	1.0	1.0	0.0001	0.0005	0.5307
		FM	1.0	1.0	1.0	0.0	0.0002	0.5744
		PL	1.0	1.0	1.0	0.0	0.0	0.0620
		PM	0.0001	0.0	0.0	1.0	1.0	1.0
		TL	0.0005	0.0002	0.0	1.0	1.0	1.0
		TM	0.5307	0.5744	0.0620	1.0	1.0	1.0
	c^{**}	FL	1.0	1.0	1.0	0.0001	0.0005	0.5307
		FM	1.0	1.0	1.0	0.0	0.0002	0.5744
		PL	1.0	1.0	1.0	0.0	0.0	0.0620
		PM	0.0001	0.0	0.0	1.0	1.0	1.0
		TL	0.0005	0.0002	0.0	1.0	1.0	1.0
		TM	0.5307	0.5744	0.0620	1.0	1.0	1.0
